# Investigating the possible effect of electrode support structure on motion artifact in wearable bioelectric signal monitoring

**DOI:** 10.1186/s12938-015-0044-2

**Published:** 2015-05-15

**Authors:** Alper Cömert, Jari Hyttinen

**Affiliations:** Department of Electronics and Communications Engineering, BioMediTech, Tampere University of Technology, Tampere, Finland

**Keywords:** Motion artifact, Surface electrodes, Textile electrodes, Un-gelled electrodes, Electrode design, Electrode structure, Wearable monitoring, Skin–electrode impedance, ECG, EMG

## Abstract

**Background:**

With advances in technology and increasing demand, wearable biosignal monitoring is developing and new applications are emerging. One of the main challenges facing the widespread use of wearable monitoring systems is the motion artifact. The sources of the motion artifact lie in the skin–electrode interface. Reducing the motion and deformation at this interface should have positive effects on signal quality. In this study, we aim to investigate whether the structure supporting the electrode can be designed to reduce the motion artifact with the hypothesis that this can be achieved by stabilizing the skin deformations around the electrode.

**Methods:**

We compare four textile electrodes with different support structure designs: a soft padding larger than the electrode area, a soft padding larger than the electrode area with a novel skin deformation restricting design, a soft padding the same size as the electrode area, and a rigid support the same size as the electrode. With five subjects and two electrode locations placed over different kinds of tissue at various mounting forces, we simultaneously measured the motion artifact, a motion affected ECG, and the real-time skin–electrode impedance during the application of controlled motion to the electrodes.

**Results:**

The design of the electrode support structure has an effect on the generated motion artifact; good design with a skin stabilizing structure makes the electrodes physically more motion artifact resilient, directly affecting signal quality. Increasing the applied mounting force shows a positive effect up to 1,000 gr applied force. The properties of tissue under the electrode are an important factor in the generation of the motion artifact and the functioning of the electrodes. The relationship of motion artifact amplitude to the electrode movement magnitude is seen to be linear for smaller movements. For larger movements, the increase of motion generated a disproportionally larger artifact. The motion artifact and the induced impedance change were caused by the electrode motion and contained the same frequency components as the applied electrode motion pattern.

**Conclusion:**

We found that stabilizing the skin around the electrode using an electrode structure that manages to successfully distribute the force and movement to an area beyond the borders of the electrical contact area reduces the motion artifact when compared to structures that are the same size as the electrode area.

## Introduction

The monitoring of various bioelectric signals, such as electrocardiograms (ECG) and the electromyograms (EMG), has already been implemented into wearable systems. One of the major issues wearable biosignal monitoring systems face is the motion artifact. A reduction in the motion artifact would be a big step towards the widespread use of dry electrodes in wearable garments that monitor physiological signals. Increased signal quality and reliability will result in systems that are not only intended for leisure and personal use but would also be suitable for monitoring for medical purposes, with better wearability and comfort.

Motion artifact reduction methods such as adaptive filtering use supplementary signals, skin–electrode impedance or motion monitoring to investigate the motion artifact after it has been generated. While being successful in reducing the motion artifact, these methods, which rely on signal analysis and the possible addition of various sensors and electronics, increase the power usage, can increase the size of the devices, and can reduce wearability.

The reduction of the motion artifact physically at its origins will provide a low cost solution that is effective and easily applicable. This reduction will translate into less motion artifact being generated overall at the biosignal input point of the system.

Studies on gelled electrodes have found that the motion artifact is mainly generated by changes in the amplitude of the intrinsic voltage source present across the epidermis [1, 2]. This voltage source is created by the injury currents and is observed as a potential difference between the layers of the skin [3]. Skin deformation by means of lateral stretching, rotational stretching, or skin stretch induced by vertical forces causes a change in this potential. Skin deformation also causes changes in the current pathways, which change the impedance, but the effect that the resulting impedance change has on the motion artifact is smaller than the effect of changes in this epidermis potential [1].

The electrical properties of the electrode gel used to create a conductive layer between the electrode and skin are stable against the movement caused by lateral and vertical forces. Since the electrode is attached to the skin by glue, the effect of the lateral forces is further reduced. The explanations given for wet electrodes may not be sufficient to explain the behavior of dry electrodes because electrode gel and glue are not used with dry electrodes. On the contrary, dry electrodes rely on a thin layer of skin moisture for increased conductivity between the electrode and the skin and do not use glue. This thin moisture layer usually forms a short time after electrode placement due to sweat [4]. As a result of motion, posture, or inappropriate electrode design, areas of non-contact or areas of varying conductivity might occur between the electrode and skin. These areas of varying conductivity will have a considerable effect on the overall electrical properties of the skin–electrode interface [5]. Furthermore, this will also cause large changes in the ionic concentrations close to the electrode, which in turn will cause changes in the half cell potential [6]. All these additional factors add up and make dry electrodes more susceptible to motion than gelled medical electrodes [7].

Unlike gelled electrodes that are glued to the skin, dry electrodes need to be secured to the skin with externally applied force. This force can be applied by a fastening apparatus or by a tight garment. In both cases, the strength of the mounting force applied to the electrodes translates as pressure applied by the electrode to the skin. This pressure causes an initial stretch of the skin and changes the initial electrical properties of the skin and the skin–electrode interface. Pressure might also cause a change in the way that the skin reacts to stretching when compared to initially un-stretched skin. Thus, the mounting force on the electrode, or the pressure applied by the electrode to the skin, might change the motion artifact resulting from similar movements [8].

Studies that compared various types of dry electrodes have found that the contact impedance changes for textile electrodes produced by different manufacturing processes such as knitting and weaving and made from different fiber material [9]. For example, signal to noise ratios differ between polymer and textile dry electrodes for EMG [10]. Also, it has been shown that there is a difference in the impedance of polymer-based dry electrodes for EMG caused by the electrode contact area [11], and that electrode area also affects noise levels in textile electrodes [12]. When a smooth electrode disc made of polysiloxane with conductive particles and a knobbed surface-structured disc made of polymer plated with Ag/AgCl are compared based on the quality of the obtained EMG signal with the signals obtained using commercial gelled electrodes, it has been reported that the performance of the dry electrodes is comparable to that of gelled electrodes [13]. In our previous study, we saw that with the same size of electrodes, the existence of a support structure between the electrode and garment that was able to act as a physical buffer reduced the motion artifact [8]. In summary, these studies show that the surface type, the materials used, or even the size of the electrode structure can have a considerable effect on the electrical properties and processes between the skin and the electrode. However, none of these studies have looked at the possible role of support structures around the electrodes in relation to the motion artifact.

In this paper, we aim to investigate whether we can reduce the motion artifact by the electrode structure design that we hypothesize to affect the origins of the motion artifact—the electrode–skin interface and the skin deformation. We investigate how these sources of motion artifact can be affected by different electrode support structures that, by design, reduce the skin and/or skin–electrode interface deformations that are caused by the motion of the subject or the direct motion applied to the electrode. To achieve comparable results, we use a previously introduced in-house designed motion artifact generation and assessment system that provides a standardized and repeatable pattern of movement applied directly to the electrode [14]. We test four different dry textile electrode support structures: a soft padding structure that extends beyond the electrode area, a similar-sized soft padding structure with a novel design intended to restrict skin deformation, and two structures that do not directly affect the skin beyond the electrode area: soft padding structure the same size as the electrode area, and a rigid support structure the same size as the electrode. The structures were tested on a standard gelled medical electrode under four mounting forces ranging from 250 to 1,000 gr and three mounting forces ranging from 1,000 to 3,000 gr. These tests are repeated for two different electrode locations to investigate the effect that tissue thickness beneath the skin has on the motion artifact.

## Methods

To investigate the relationship between applied motion and the motion artifact for the electrodes, we subjected the electrodes to programmable and controlled motion under monitored mounting force using our novel motion artifact generation and assessment system. As the motion was applied, we simultaneously measured electrode–skin impedance and the surface biopotential from the electrodes. Overall, six different electrode mounting forces were applied and the effectiveness of the support structures for differing tissue properties was investigated by testing the electrodes on two separate anatomical locations. The obtained data were analyzed in time and frequency domain for comparison of the functioning of the electrode support structures for each mounting force and for different tissue properties.

The motion artifact generation and assessment system comprised a module for the motion generation and a module for data acquisition as described in [14]. In short, the lateral motion of the electrode was created by a heavy duty, high torque hexTronik HX12K Hi-speed servo (Hextronik Limited, ChenDu, China) that was mounted on the vertically movable platform of a Dremel 220 workstation (Robert Bosch Tool Corp., IL, USA). The servo was controlled by an Arduino Uno microcontroller (SmartProjects, Turin, Italy), which can be programmed via a PC. A FlexiForce (Tekscan Inc., South Boston, USA) sensor was situated between the electrode and the servo to monitor the mounting force exerted on the electrode. As this sensor is non-linear, its output was linearized using op-amp circuitry, as suggested by the user guide of the sensor, before being fed into the Arduino Uno microcontroller that processes the reading and provides feedback to the user about the magnitude of the exerted force by lighting up the appropriate LEDs.

**Figure 1:**
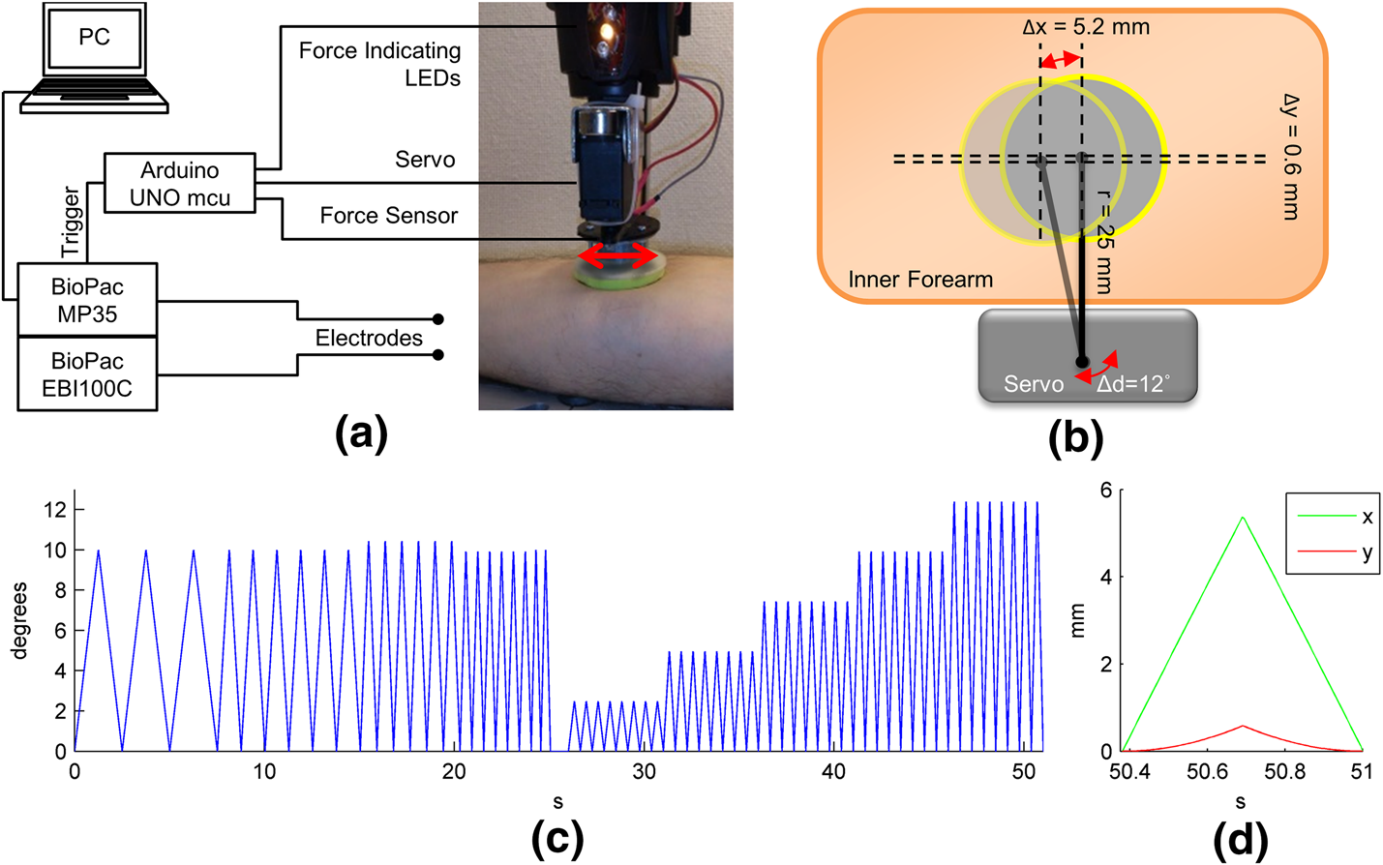
System overview of the motion artifact creation and assessment system, and the servo rotation pattern used to create motion at the electrode. **a** Presents the system overview and for simplicity, the electrode locations are not shown. **b** Translation of servo rotation into lateral electrode movement. **a** and **b** are modified from [14] and the applied motion is depicted by the *two-sided arrows*. **c** Rotation of the servo in degrees. **d** Translation of this pattern into lateral electrode motion in x- and y-axes.

The data acquisition and analysis system comprised a BioPac Data Acquisition Unit main module MP35 for measuring the motion artifact and the ECG, and the bioimpedance module EBI100C for simultaneously measuring the skin–electrode impedance (BioPac Systems, Inc., CA, USA). As the motion was applied, three measurements were made simultaneously: the skin–electrode impedance measurement, a biopotential measurement that was almost purely motion artifact, and a biopotential measurement that contained both the ECG and the motion artifact. The sampling rate for these measurements was 200 Hz. The electrode skin impedance was measured using a 100 µA current with 100 kHz frequency. Even though this current frequency is much higher than the frequency bands covered by the motion artifact and the ECG, the skin–electrode interface impedance changes due to motion can be clearly observed at this current frequency [15]. An overview of the system is presented in Figure 1a.

The motion of the electrode was generated with a rotating servo motor that is connected to the electrode via a 25 mm long servo arm. The displacement of the center point of the electrode at the largest servo rotation, as explained below, was 5.2 mm in the x-axis and 0.6 mm in the y-axis, an almost linear back and forward motion. This translation of the rotational motion of the servo to the lateral motion of the electrode is presented in Figure 1b.

We used a rotation pattern that was divided into two parts. For the first 25-s, we increased the speed of the motion while keeping the displacement almost constant. This motion pattern was designed to reveal the effect of changing frequency on the motion artifact. After a 1-s pause, the second motion pattern, comprising an increase in both speed and displacement amplitude, and thus constant frequency, was started. This motion pattern was selected to give an understanding of the effect of increased motion magnitude. The rotation pattern is presented in Figure 1c. The translation of servo rotation to electrode displacement is presented in Figure 1d, where one sample rotation for each rotation magnitude is presented.

**Figure 2:**
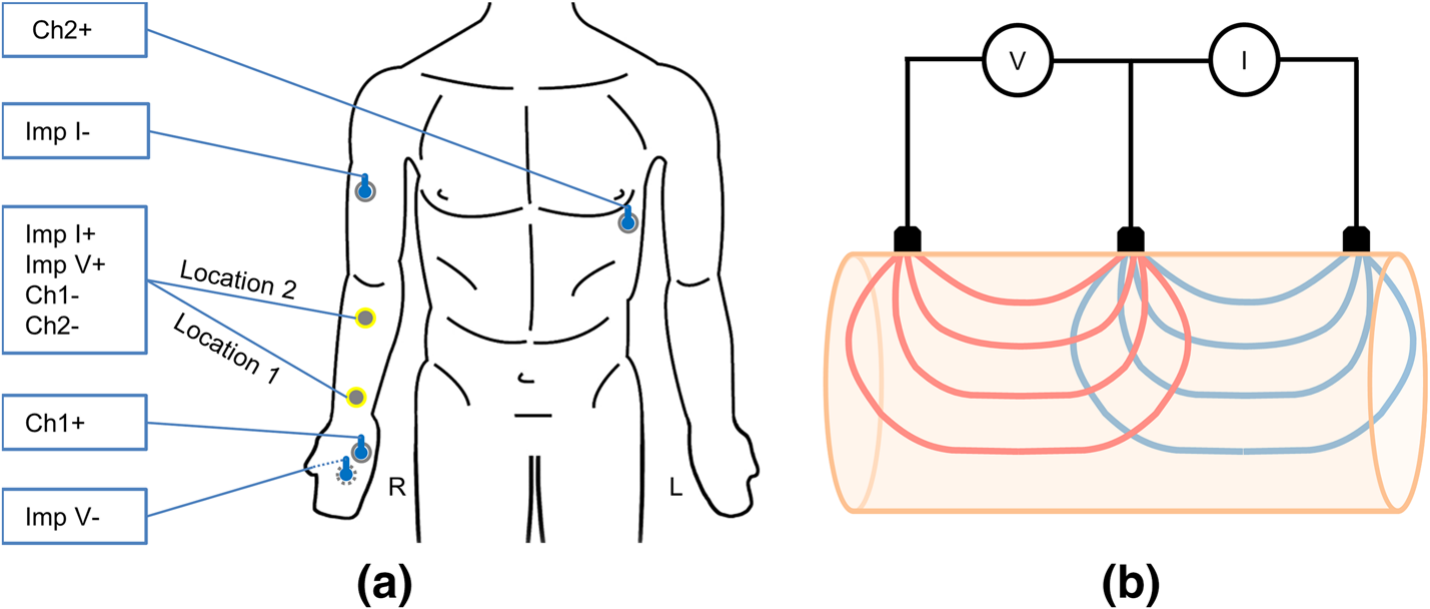
The electrode setup and the sensitivity field of the three electrode impedance measurement setup. **a** The electrode setup. Textile electrodes are shown as a *gray* *centerpiece* with *yellow outline*, and the medical electrodes are shown as a *blue* *centerpiece* with *gray* *outline*. Please note that for experiments with Electrode M, explained below, the textile electrode locations are occupied by medical electrodes. **b** The sensitivity field of the three electrode impedance measurement setup. Due to reciprocity, these two fields can be used interchangeably for voltage and current, and the sensitivity field is defined as the area covered by both fields [20]. The presented figure is different than the sensitivity distribution of the impedance measurement. The sensitivity distribution is the dot product of the current density fields of the current feeding electrodes and voltage measurement electrodes. The dot product of these vector fields is a scalar and is maximum when the fields are parallel, zero when the fields are perpendicular, and negative when the angle between the fields is larger than 90° [21, 22]. In the presented figure, the areas impacting the measurement are the intersection areas of the two fields.

To investigate the impedance changes caused by electrode motion, the biopotential changes caused by electrode motion, and the ECG affected by this motion artifact, three simultaneous measurements were carried out using the electrode subject to motion in all three measurements leads, as shown in Figure 2a. To investigate if tissue differences cause a change in the system’s susceptibility to motion artifact or a change in the functioning of different electrode structures, we used two different locations for the electrode subject to motion. The first location was on the inner forearm, 5 cm proximal to the wrist, with thin tissue between skin and bone. The second location was also on the inner forearm, 5 cm distal from the elbow crease, with thick forearm muscle tissue beneath the electrode location. The motion artifact was measured between the electrode subject to motion and the inner palm, a configuration that has only minimal intrusion of ECG. The ECG containing the motion artifact was measured between the electrode subject to motion and an electrode at the V5 location. The impedance measurement was done in a three-electrode setup, with high sensitivity at the electrode–skin interface. The current injection and positive voltage was set to be at the electrode subject to motion, the current sink electrode was located on the outside of the upper arm, and the negative voltage electrode was located on the outer wrist close to the 4th and 5th digits. The sensitivity field of this measurement setup is presented in Figure 2b. As can be seen in Figure 2b, the main volume measured by the three-electrode setup is the skin electrode interface and the immediate tissue under the common electrode area. The three-electrode setup also measures the underlying deeper tissue. However, this cannot be avoided by any electrode systems when the electrode impedance is measured on skin. Thus, we consider the three-electrode setup as optimal for measuring the electrode impedance changes generated at the skin–electrode interface caused by electrode movement.

**Figure 3:**
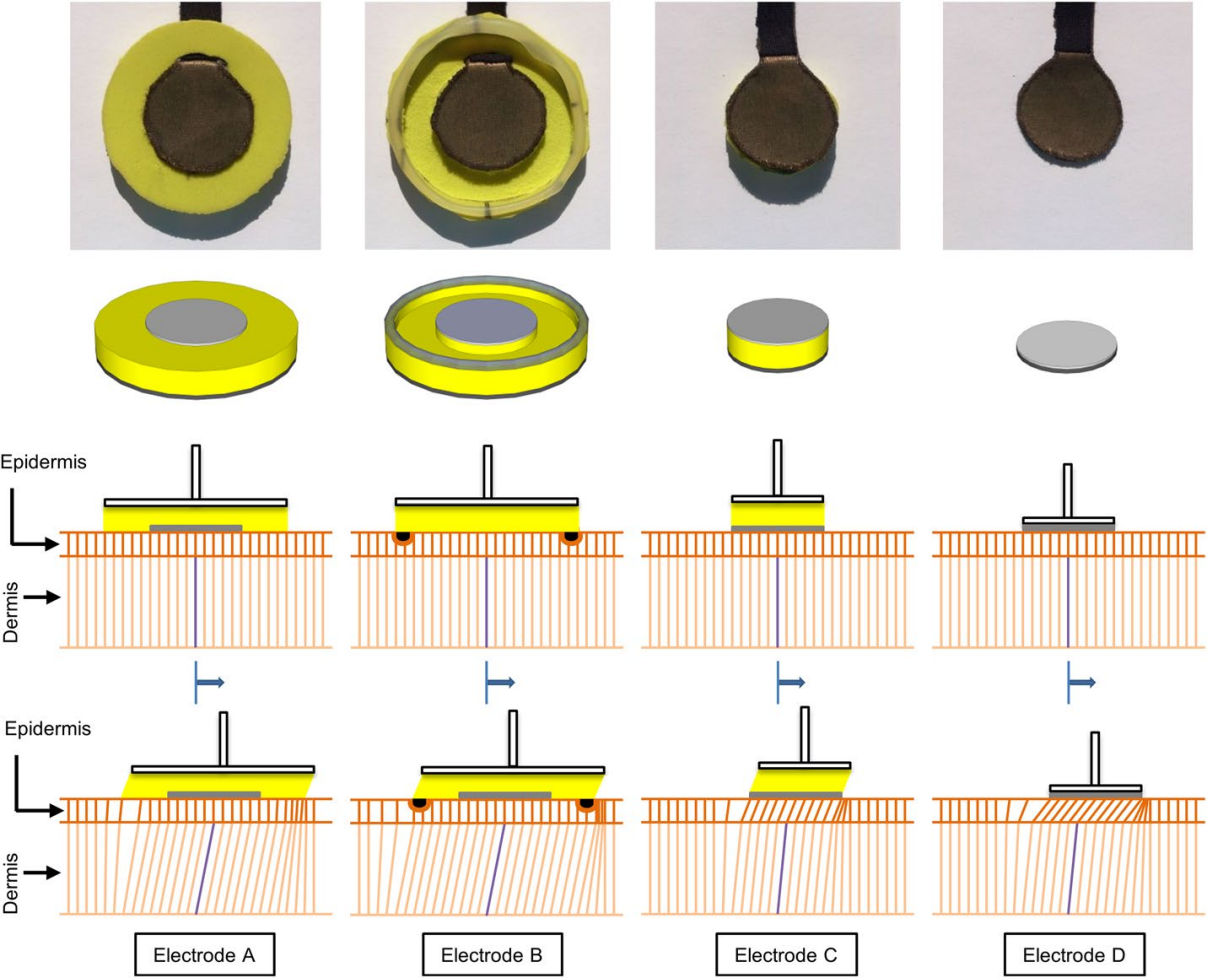
Photographs and concept models of the electrodes and the proposed physical effect of electrode structure on the skin layers. Electrode A comprises a textile electrode supported by 40 mm diameter padding. Electrode B has an 8 mm wide, 2 mm deep ridge carved out of the 40 mm diameter support padding starting at the edges of the electrode and finishing 2 mm before the outer edges of the padding. A 2 mm thick, 1 mm high silicon ring has been placed on the outer edge. Electrode C has the textile electrode supported by padding the same diameter as the electrode. Electrode D has the textile electrode attached directly to the hard plastic of the electrode to the servo connector part. In the concept model drawings of the *second row*, the *top grey circle* is the textile electrode surface, *yellow* is the soft padding, and the *bottom dark grey* cylinder is the plastic connector part to the servo. In Electrode B, the *grey ring* is the outer silicon ring mentioned above. In the *third* and *fourth rows*, the proposed physical effect of the electrode structure on the epidermis and the dermis is presented. The epidermis is depicted in *orange* and the dermis is depicted in *pink*. The *center blue line* in the dermis is to provide an easier comparison between the drawings. The *black dots* under Electrode B are the cross-sectional view of the epidermis-constricting ring at the outer boundary, as shown in. We expect this narrow ring to dig into the epidermis. The larger electrode, because of the larger amount of epidermis displaced, might force the lower levels of the skin to also displace resulting in the epidermis being displaced less and thus causing a smaller motion artifact.

Four different dry electrodes with different structures supporting the electrode textile and one commercially available gelled electrode were tested in our experiments. These electrodes are illustrated in the first and second rows of Figure 3. The textile electrodes were made of MedTex P130 silver-coated yarn textile (Statex Productions and Vertriebs GmbH, Bremen, Germany) and had a diameter of 20 mm. The paddings used in the experiment were made from Poron Impact Cushion (Rogers Corporation, Rogers, USA) with a thickness of 4 mm. Electrode (A) had a support cushion 40 mm in diameter. Electrode (B) had a 40 mm diameter support cushion with a unique structure: an 8 mm wide, 2 mm deep ridge was carved out of the support cushion starting at the edges of the electrode and finishing 2 mm before the outer edges of the padding. Then, a 2 mm thick, 1 mm high silicon ring was put on the resulting outer padding border. Electrode (C) had a support cushion 20 mm in diameter that did not extend beyond the electrode area. Electrode (D) had no padding and was connected to the hard plastic of the mounting system, providing the electrode with a support structure that was motionless and rigid. Electrode (M) was a commercial Ambu Blue P brand medical electrode (Ambu A/S, Copenhagen, Denmark).

From our preliminary investigations, we assumed that a soft support cushion acts as a buffer between the skin and the motion source, like a spring suspension in three dimensions [8]. Guided by earlier studies that reported that the main motion artifact is cause by the change in the potentials across the epidermis in response to deformation [2, 3, 16] and that this potential change decreases further from the deformation [17], we propose that a padding larger than the electrode surface might reduce the epidermis deformation directly under the electrode by distributing the deformation over a larger area of the epidermis and by moving the border of the movement away from the electrode. We further suggest that by restricting the deformation of the epidermis by using a constraining structure such as Electrode B this effect can be increased. In this case, the epidermis, which is a fraction of a millimeter thick, might be held in place by the outer ring of the electrode and the electrode movement that would result in epidermis deformation might be effectively transferred to the dermis. This would still result in skin deformation, but at a layer that can be simply modeled as a resistor and will not generate potentials at the level of the conventional motion artifact amplitudes [6]. In the third and fourth rows of Figure 3, we present this idea as a comparison between electrode structures in an exaggerated way for a clearer description.

## Experimental procedure

Before each experiment, the electrode mount force measurement was calibrated using a Soehnle Siena kitchen scale with an accuracy of 1 gr (Leifheit AG, Nassau, Germany). The electrodes were moistened with four drops of tap water to simulate the presence of sweat. It was observed that the moistness remained stable throughout the duration of the experiment. The stability of the conductive layer between the skin and the electrode was also observed to have stable impedance levels throughout the experiments. Therefore, reapplication of moisture was unnecessary. An important factor here might be that the skin under an electrode starts to perspire a few minutes after electrode application [7]. The electrode was mounted on the experiment location with the experiment-specific starting mounting force and the pre-programmed motion presented in Figure 2 was applied. At the same time, the skin–electrode impedance, the motion artifact, and the ECG with the motion artifact were measured. When the motion pattern finished, the applied mounting force was increased to the next level and the process repeated without lifting the electrode. When the final force level for that experiment was reached, the electrode was lifted from the skin completely and remounted at the same location. The experiment was then started again with the lowest force. For Electrodes A, B, and C, three such procedures were carried out for each electrode for each location. For the medical electrode, repeating the procedure once was thought to be adequate as we were more interested in textile electrode behavior. Furthermore, the behavior of medical electrodes has been extensively studied, and in our previous research medical electrodes were found to be relatively stable against motion artifact. Because of complaints from the volunteers about the discomfort caused by the rigid support of Electrode D, only one repeat of the procedure was done for this electrode.

Two separate experiments were done. The first experiment investigated mounting forces of 1,000, 2,000, and 3,000 gr. The second experiment looked in more detail at low to moderate mounting forces by using 250, 500, 750 and 1,000 gr of mounting force. Due to system design, device connectivity and synchronization issues were encountered between the Arduino and the BioPac. As a result, we were only able to monitor the initial application force. The force changes during motion application were not monitored. Five volunteers participated in both experiments. The impedance and the motion artifact data were filtered between 0.2 and 10 Hz to highlight the signals coming from the motion and to reduce higher frequency noise and baseline drift in the lower frequencies which were not caused by the applied motion. These filters are much narrower than those used for heart-rate monitoring. For the monitoring of heart rate, it is adequate to have filters with a pass-band of 0.5–40 Hz and the 0.05–150 Hz bandwidth is set as a requirement for diagnostic applications [18]. It has been proposed that these low frequency criteria be eased for diagnostic applications [19]. The reason for our filter selection was that we were interested in the motion artifact superimposed on the relevant signals and not ECG or EMG analysis. Our prior experiments had shown that the motion artifact created by our motion generator stays within the frequency bands of the applied motion (data not shown) [14, 15]. This observation has also been confirmed by the results presented in this paper. The resulting filtered data were visually analyzed, and for numerical analysis the root mean square amplitude (RMS) of the motion artifact and the RMS of the impedance change were used for noise comparison. The data obtained during the second part of the motion pattern of Figure 1c were divided into five windows, each corresponding to one movement magnitude. The RMS values of this data were used to investigate the effect of movement magnitude on the motion artifact for the different electrode designs. The power spectrum densities (PSD) of the data were calculated and compared with the PSD of the preprogrammed motion to assess the similarity between the applied motion, the impedance, and the motion artifact.

## Results

**Figure 4:**
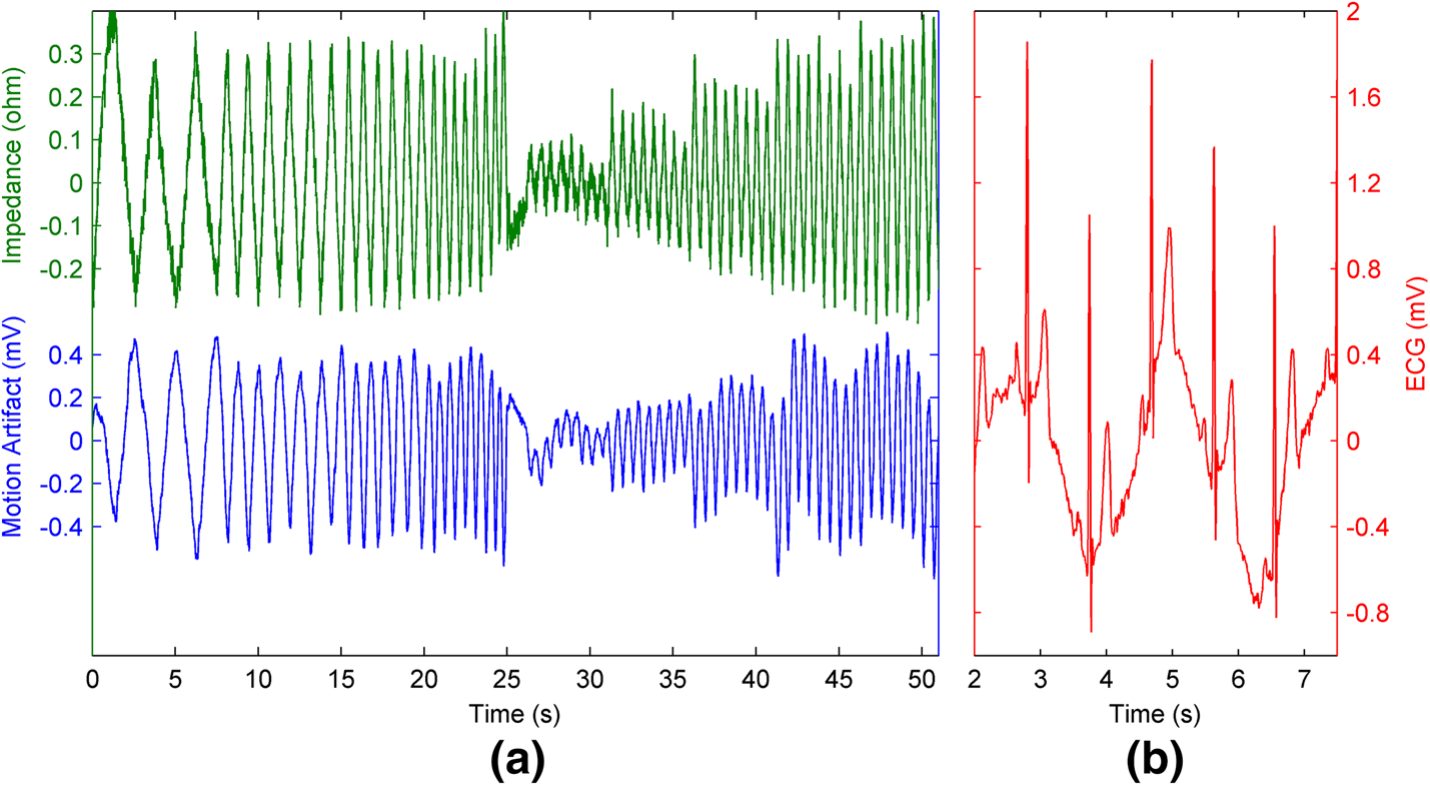
Example of skin–electrode interface impedance and the motion artifact signal of one experiment session, and a close up of the ECG during the same session. **a** The first 25-s of the graph shows the effect of increasing the speed of motion while keeping the amplitude almost constant (see Figure 1). After a 1-s pause, the second 25-s segment shows the effect of motion with amplitude and speed increasing in the same ratio. **b** The ECG affected by the motion artifact. The y-axis scale of the ECG and the motion artifact in are same while the scale of the x-axis of the ECG has been increased by a factor of 4 relative to the x-axis of the motion artifact to better present the ECG components. The signals are filtered between 0.2 and 40 Hz.

The skin–electrode interface impedance change caused by electrode movement, the motion artifact, and the ECG affected by the motion artifact are presented in Figure 4. As can be seen Figure 4a, the skin–electrode interface impedance and the motion artifact are closely related. Furthermore, the main frequency components of the signals are similar with the exception of a low frequency component in the motion artifact. The frequency change of the motion in Pattern 1 and the effect of increasing the movement magnitude in Pattern 2 can be clearly observed in the resulting signals. Figure 4b presents the distortive effect of the motion artifact on the ECG.

**Figure 5:**
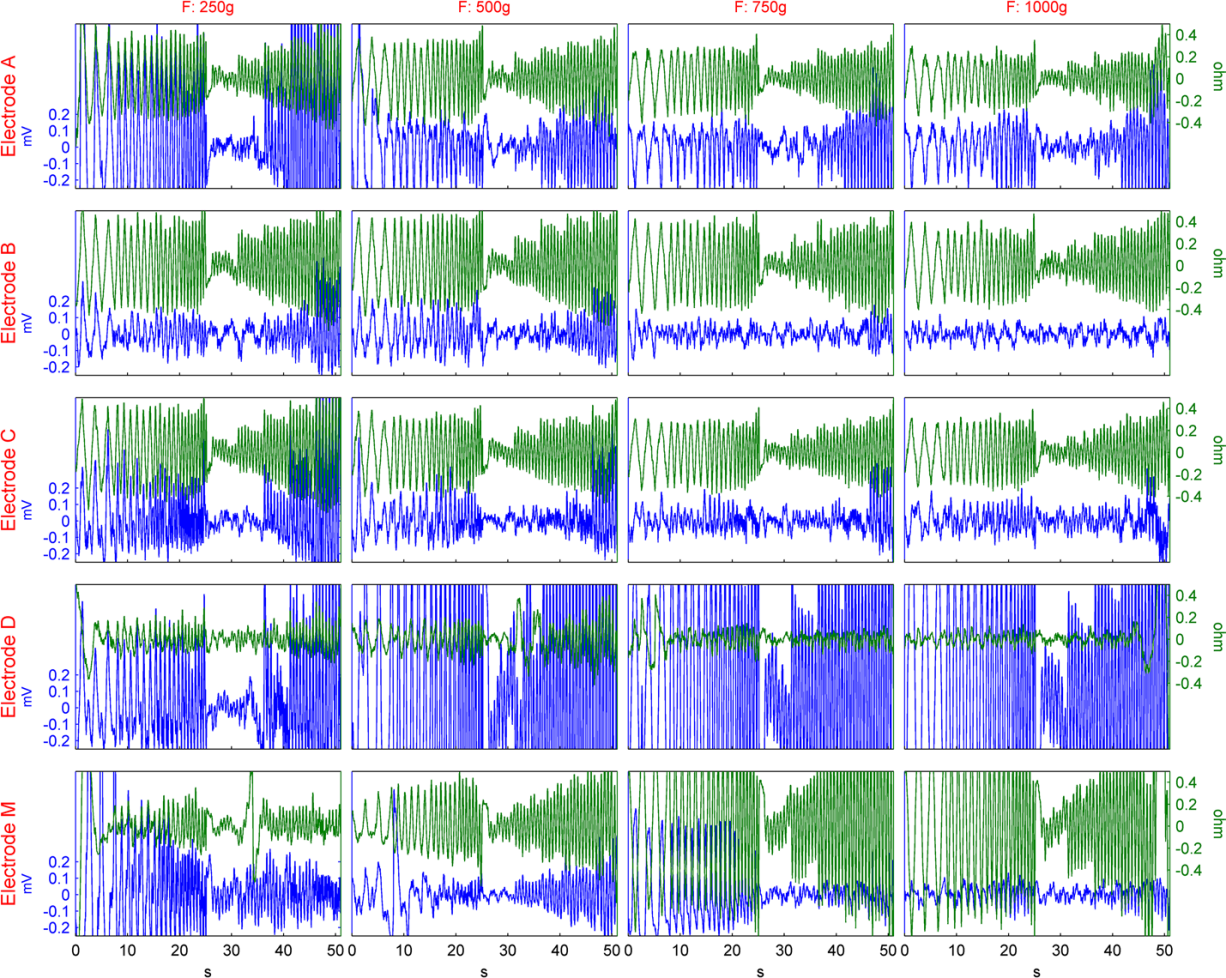
Example of data from one subject, measured from the distal forearm location. Electrodes are presented in *rows* and the applied forces are arranged in *columns*. To gain space for clarity, the *scales* in the inner axes are not shown.

In Figure 5, we present a sample of the original data of all the electrodes and forces of Experiment 2 for one subject measured from the distal forearm location. The difference between the electrode structures in response to motion is observed. The stabilizing effect of soft padding can be seen between Electrode D and Electrodes A, B, and C in Figure 5. The decrease in impedance change and the motion artifact with increasing force can also be noticed. The relationship between impedance change and motion artifact, which was observed in Figure 4, is present, yet Figure 5 also shows the lack of a relationship between the peak amplitudes of these two signals.

**Figure 6:**
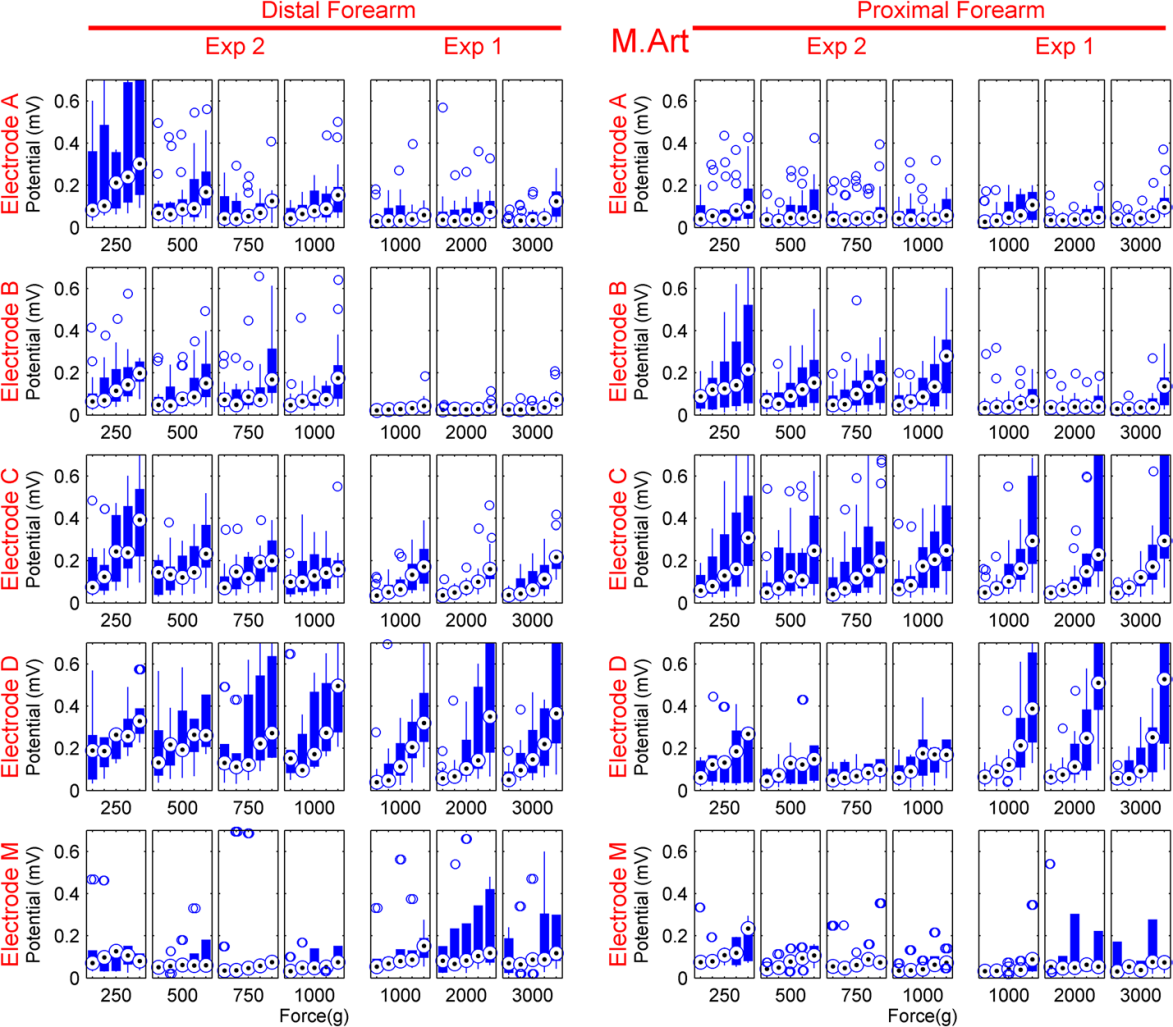
The RMS amplitudes of the motion artifact calculated for each motion range of Pattern 2 presented as *boxplots*. Each *box* corresponds to a specific applied force and the motion artifact RMS’s are plotted for each increasing range of Pattern 2 from *left* to *right* (2.5°, 5°, 7.5°, 10°, and 12.5° of servo rotation). The *dot* in the *center* of *each* *blue column* is the median, and the *edges* of the *blue columns* are the 25th and 75th percentiles. The *lines* that extend from the *columns* are the data points out of this percentile range which are not considered outliers. The outliers are plotted separately as *empty circles*.

The RMS median and 25th and 75th percentile values of the motion artifact for increasing movement magnitude, covering all subjects are presented in Figure 6 as boxplots. The median values of a dataset are presented as the central dots in the columns, while the columns present the data falling within the 25th and 75th percentile range. The medians and the range that the data falls within for a given electrode and condition displays an observable trend in electrode behavior. The data correspond to Pattern 2 and are separated into five sections, one for each rotation amplitude. Each electrode is presented in one row. First Experiment 2 and then Experiment 1 are presented consecutively for both locations. The two columns on the left are for the distal forearm location and the two columns on the right are for the elbow location. For ease of comparison, all graphs have the same scale. The 75th percentile lines corresponding to the high values obtained from the fourth electrode were omitted for a better qualitative comparison. An overall idea of the motion artifact for a given electrode for the specific experiment condition can be gained from these plots. For forces up to 1,000 gr, the lowering and stabilizing of the motion artifact is observed for the soft padded electrodes. The advantage of using flexible padding between the electrode and the force applied mounting system over using a rigid support structure between the electrode and the applied force can be clearly seen in the wrist location where the tissue between bone and skin is very thin. It can also be observed that increasing the angle increases the created motion artifact, and that there is a disproportional increase in the slope at the largest movement. This increase might be due to the induced skin stretch getting close to the elastic limits of the skin. The effect of electrode structure on this behavior can also be recognized.

Table 1 presents the medians of the RMS amplitudes of the motion artifact and the standard deviation of these amplitudes (in parentheses) for each electrode for selected applied force levels at each location. In Table 1, a summary of the data in Figure 6 is presented and serves as an estimation of how each electrode would function in a tight garment, and how this tightness could affect the motion artifact. For a more detailed understanding, the reader is kindly directed to Figure 6. The data is taken from all subjects of Experiment 2, and selected so that the applied force corresponding to the data was at user-friendly levels that create good electrode–skin contact, namely 500, 750, and 1,000 gr. As a result, it was possible to make a comparison of the electrodes under the most likely garment tightness conditions. The RMS amplitude is calculated from the complete 51-s window of each measurement session. In the distal location, Electrode A and B have the least motion artifact among the textile electrodes as well as low variability. The generated motion artifact amplitudes remain stable throughout the changes in applied force. The medical electrode has the lowest median amplitude in this location, but increasing the force acting on the electrode introduces a large variability to the amplitudes, and makes this electrode unpredictable in its behavior. In the proximal location, Electrode A has the lowest motion artifact, again with low variability. Electrode B has a higher motion artifact in the proximal location than in the distal location, on par with Electrode C and D, but has a much lower variability than the latter two. In the proximal location, the medical electrode has similar motion artifact amplitudes as in the distal location, yet with considerably smaller variability. The low median amplitude of the motion artifact of the medical electrode is only valid for the force levels presented in Table 1, and it can be observed from Figure 6 that the motion artifact of medical electrodes quickly increases at higher applied forces. Furthermore, Figure 6 also shows that the motion artifact of Electrode A and B is decreased even further at the higher mounting forces: to levels lower than the median amplitudes of the motion artifact of the medical electrode at any given mounting force. Thus, Electrode A and B are found to be the best electrodes overall.

**Table 1:** Median values and the standard deviation of motion artifact RMS amplitudes for electrodes mounted with user-friendly mounting forces

	Distal forearm location			Proximal forearm location		
	Median (mV), [STD (mV)]			Median (mV), [STD (mV)]		
	500 gr	750 gr	1,000 gr	500 gr	750 gr	1,000 gr
Electrode A	0.12 (0.15)	0.08 (0.07)	0.12 (0.06)	0.07 (0.08)	0.06 (0.07)	0.05 (0.06)
Electrode B	0.11 (0.10)	0.11 (0.14)	0.13 (0.17)	0.17 (0.10)	0.14 (0.09)	0.19 (0.10)
Electrode C	0.21 (0.08)	0.17 (0.08)	0.16 (0.08)	0.17 (0.14)	0.12 (0.19)	0.18 (0.25)
Electrode D	0.22 (0.29)	0.19 (0.25)	0.38 (0.19)	0.14 (0.22)	0.08 (0.55)	0.16 (0.47)
Electrode E	0.07 (0.17)	0.08 (0.27)	0.09 (0.43)	0.11 (0.05)	0.08 (0.05)	0.06 (0.05)

**Figure 7:**
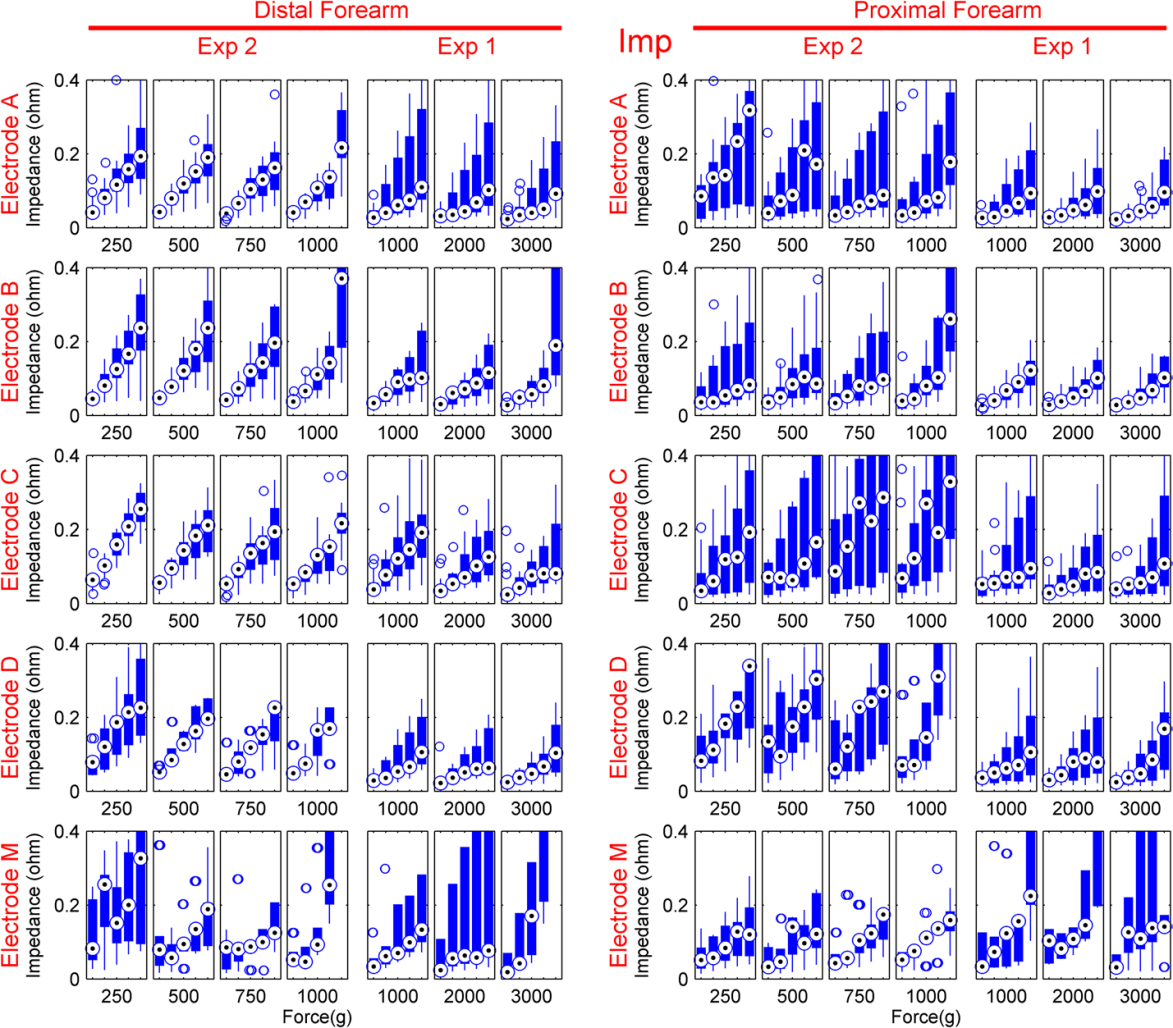
The RMS amplitudes of the change in the impedance calculated for each motion range of Pattern 2 presented as *boxplots*. Each *box* corresponds to a specific applied force and the motion artifact RMS’s are plotted for each increasing range of Pattern 2, from *left* to *right* (2.5°, 5°, 7.5°, 10°, and 12.5° of servo rotation). The *dot* in the *center* of *each* *blue column* is the median, and the *edges* of the *blue columns* are the 25th and 75th percentiles. The *lines* that extend from the *columns* are the data points out of this percentile range which are not considered outliers. The outliers are plotted separately as *empty circles*.

The changes in the impedance related to the movement amplitude are shown in Figure 7. As with the motion artifact, the impedance change also increases with increased movement; the linearity of this increase is seen to depend on the electrode structure and the location, and varies greatly. The measured impedance had a median of 83.1 Ω throughout the experiments.

**Figure 8:**
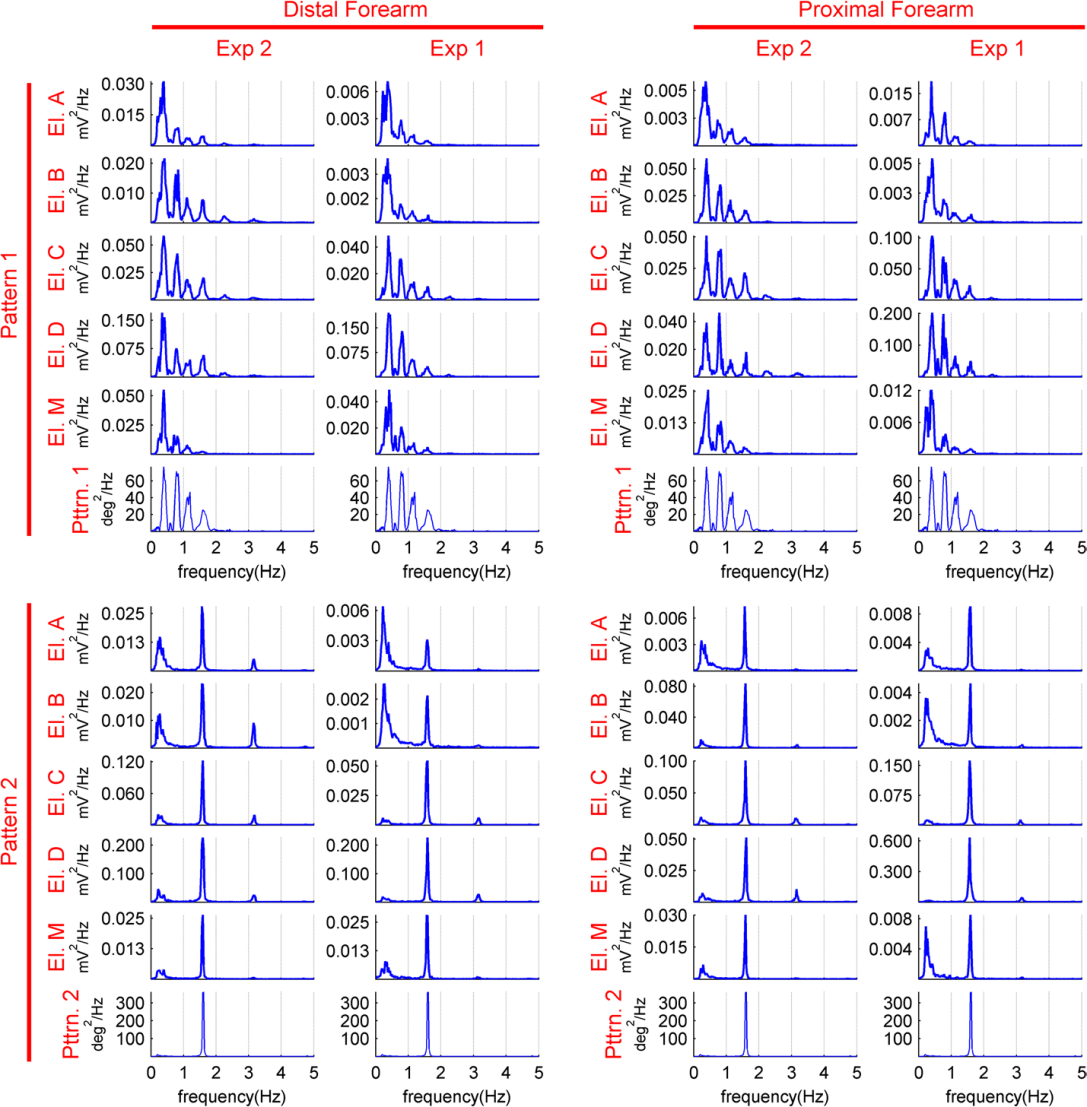
The medians of the PSD’s of the motion artifact. The PSD’s are shown from 0 to 5 Hz and the data is filtered between 0.2 and 10 Hz. The *first group of* *rows* shows data from experiments with Pattern 1; the *second group of* *rows* shows data from experiments with Pattern 2. The *first group of* *columns* represents experiments at the distal location; the *second group of* *columns* represents experiments at the proximal location. Each PSD graph is presented with its own scaling to make comparison easier.

The medians of the power spectrum densities of the motion artifacts for each electrode for each experiment are calculated for all subjects and forces. The data are presented in Figure 8. The presence of the frequency components of the applied motion pattern is seen throughout the motion artifacts. This is clear in the Pattern 2 segment of the graphs, as Pattern 2 has a single peak at 1.5 Hz. We wanted to preserve the spectrum form in each case. Thus, the reader is advised to pay attention to the scales of the plots before comparing the presented data. In some cases such as when comparing the PSDs of the motion artifact of Electrode B at the proximal location corresponding to the lower mounting forces of Experiment 2 with the PSDs of the same setup under the higher mounting forces of Experiment 1, an increase of signal power in the low frequencies is observed. However, this noise component is similar in all electrodes. On a related note, the differences between electrode behaviors under lower applied force and higher applied force can be seen in Figure 8. Though not investigated, the difference in electrode behavior regarding the movement frequency can be observed from the Pattern 1 graphs.

The frequency spectrum of the impedance change is more closely related to the frequency spectrum of the motion pattern than is the case with the frequency spectrum of the motion artifact presented in Figure 8. The low frequency noise observed in the motion artifact frequency spectrum for the textile electrodes is negligible in the frequency spectrum of the impedance change, making the frequency components of the motion pattern directly observed in the frequency components of the impedance change.

## Discussion

Our results clearly show that by proper design of the electrode support structure we can reduce the severity of the motion artifact. The effect of the structure design is seen both in the measured surface potential as motion artifact and the change in the skin–electrode impedance, both of which follow the applied motion pattern. This study adds to our previous research [8] in which we reported the positive effects of using a padding as support structure between the electrode and garment and points to the importance of the design of this structure. It is important to note the support structure dimensions need not be limited to the electrode size.

The two larger electrode padding designs, the simple structure of Electrode A and the skin constricting structure of Electrode B with a stability ring, work better than the smaller padding of Electrode C in all cases. The hard support of Electrode D presents high movement artifact in the distal forearm location, but, to our surprise, had low movement artifact in the proximal location. The motion artifact of Electrode A and B is comparable to and even lower than the standard medical electrode, Electrode M.

There is a clear difference in electrode behavior between the distal forearm location and the proximal forearm location. In the distal location, close to the wrist, there is very thin tissue between the skin, the bones, and the tendons. Because tendons are also harder than muscle tissue or fat, they can be thought of as being hard material between the skin and bone. In the proximal location, the inner forearm close to the elbow crease, thick muscle and in some cases fat tissue exists between the skin and underlying bone tissue. When the soft tissue between the skin and the underlying harder structures is thin, the use of padding between the applied force and the textile electrode considerably dampens the motion artifact compared to a rigid support structure (Electrode D). This is due to the softer support allowing the skin and electrode layers to be pressed in unison onto the bone and tendon structure. In other words, the padding allows the electrode to take the shape of the more rigid anatomical structures below. The harder the support structure is, i.e. in our case the exclusion of soft padding, the more it forces the electrode surface to keep the support structure’s rigid shape. This affects the forces acting on the skin depending on the anatomical structure below and causes the electrode to exert more pressure on the skin location where there is tendon or bone underneath and less pressure when there is only soft tissue underneath the location. Thus, the electrode skin contact is less homogenous, and might even cause non-contact areas to occur. On the other hand, for the proximal electrode location, the use of the rigid structure (Electrode D) resulted in a smaller motion artifact than was obtained from the use of the smaller padding (Electrode C) on this soft tissue. The reason for this could be that the pressure exerted on the skin by the rigid border of Electrode D is higher and concentrated on the border, while the pressure exerted by the softer and deformed borders of Electrode C is distributed along the edges of the soft padding. This might cause the skin under Electrode D to be more restricted in its mobility than under Electrode C. Thus, reducing the motion artifact. Electrodes A and B, with the larger paddings, possessed a similar motion artifact for both locations. Here it is important to note that because only one repeat per experiment was done for Electrode D, the results presented are not statistically supported.

These results point to the possibility that the hypothesis we presented in the third and fourth rows of Figure 3, in which we postulate that an electrode that minimizes epidermis deformation in response to motion by a skin constricting design will effectively reduce the motion artifact at its source, has potential and should be investigated further. However, modifications that take into account tissue properties and electrode pressure still need to be done. Overall, the idea needs more study to be proven right or wrong.

Regardless of location, the mounting force seems to reduce the motion artifact up to 1,000 gr. This is seen especially in the low force range: a low force of 250 gr cannot reliably secure the electrode on the skin and generates large motion artifacts in all textile electrodes. The next applied force of 500 gr can secure the connections and increasing the force lowers the motion artifact up to 1,000 gr. After that, the effects of increasing the force produce uncontrolled results. It is important to note that due to the difficulty of reliably measuring the pressure under electrodes of different sizes, support structures of different elastic properties, and for the differing anatomies of the volunteers we measured the force applied to the electrode. The pressure applied by the electrode to the skin is inversely proportional to the surface area of the electrode. Consequently, the pressure applied to the skin by Electrodes C and D are approximately 4 times higher than Electrodes A and B for the same applied force. The forces applied on Electrodes A and B for the first experiment (1,000, 2,000, and 3,000 gr) produce the same pressure applied to the skin as for Electrodes C and D in the second experiment (250, 500, and 750 gr). Thus, even if Electrode D provides a good motion artifact response on the experiment 1 forces equivalent to 1,000–3,000 gr applied weight, it loses out on the user comfort side to Electrode A and B due to the high pressure under the electrode. Electrode D seems to be very uncomfortable on the electrode borders caused by the rigid structure. This points to the importance of garment design and material selection because the tightness of the garment changes due to changes in anatomical shape during body movement and muscle contractions. As a result, the force applied to the electrode by the garment is affected.

Our analysis of the impedance and the motion artifact in relation to the motion magnitude shows that generally the motion artifact increases gradually for smaller movements. For larger deformation, however, the motion artifact signal is more amplified than the movement. This may originate in the limit of skin elasticity being reached, and, as a result, the motion artifact gets worse. It is obvious that after the limits of skin elasticity have been reached and surpassed, the electrode will dislocate on the skin and make the signal obtained useless. Interestingly, the rise in impedance can be said to be linear for padded textile electrodes.

In addition to the clear motion artifact due to the applied motion, the motion artifact has both low frequency noise content with small amplitude and a baseline drift not caused by our experiment setup and methodology. On the other hand, the impedance change does not possess high frequency noise and the signal is very closely related to the applied motion.

In frequency domain analysis, it can indeed be seen that the measured surface potentials contain the motion artifact frequency components that coincide with the applied motion pattern. The low frequency noise and the baseline drift can be clearly observed in the PSD’s of the surface signals that have a motion artifact of lower amplitude. For these signals, the low frequency noise and the baseline drift are higher not in amplitude but larger relative to the motion artifact. We think this low frequency noise and the baseline drift are caused by factors related to heartbeat, breathing, and very low level, uncontrollable muscle activity being picked up by the electrodes.

The PSD graphs of the impedance change also show the direct relationship between the applied motion and the caused impedance change. As in time domain, this relationship is clearer than it is for motion artifact and confirms the time domain observation. In this paper, we concentrate on the motion artifact measured as a surface potential, but would like to add a few words on the impedance change induced by motion. We already mentioned the similarity between the measured impedance change and the applied motion pattern, which is very clear even if the change in impedance amplitude is less than 1% of the absolute impedance measured in our three electrode setups presented in Figure 4. Even with this clear relationship between the impedance change and the applied motion pattern, there is no relationship between the peak values or the RMS values of the impedance change and the peak values of the RMS values of the motion artifact. The magnitudes are not related, but the presence of the motion artifact and the frequency components of the motion artifact can be deducted from the impedance changes. This would suggest that electrode skin impedance could be used as a predictor of the presence of motion artifact and as an input parameter for motion artifact reduction algorithms. However, the procedures should take into account these nonlinear relationships.

In the initial stages of wearable monitoring system development, electrode design was seen as being separate from garment design. However, because these two parameters together reduce the motion artifact, both are crucial. Using the skin deformation mechanism under the electrode, guidelines for the design of motion artifact-resistant dry electrodes can be developed. Such guidelines will help in the wider and more reliable use of wearable monitoring devices.

## Conclusion

We have shown that physical electrode structure design and how it supports the skin is an important factor in dealing with the motion artifact. Our results show that a support structure that restricts epidermis deformation in response to motion and/or distributes/relocates/or extends the movement applied to the skin beyond the electrode surface has potential to reduce the motion artifact at its origins. Most probably, the good performance of the medical electrode is partially due to the same phenomena as the glued surface around the gelled electrode acts in a similar manner.

The anatomical properties of the skin and the underlying tissues at the electrode location have a profound effect on the performance of the electrode and electrode structure as well as on the severity of the generated motion artifact. However, we see the possibility that a well-designed electrode can minimize this effect and have good functionality independent of electrode location and skin and tissue properties.

Nevertheless, the electrode, its support structure, and garment design should be taken as an integrated design task. All these structures together need to be designed to keep the movement of the electrode to a minimum and have adequate tightness and support for skin stability. This is especially important considering that the motion artifact increases out of proportion to the applied motion for larger electrode movements. As presented, the force applied to the electrode affects the motion artifact, and makes it necessary for the garment to be designed to minimize the effect of tightness changes on the motion artifact. In this context, one advantage of using padding is that it creates a bulge on the inside of the garment, and the increased pressure under the bulge reduces the tightness criteria that are required for adequate electrode contact pressure for the rest of the garment.

In a previous study using electrodes implemented in garments, we found that using padding was always better than the electrode surface being placed directly on the garment textile [8]. In this study, taking into account usability and comfort, we see that using soft padding is better than using a rigid support structure. Furthermore, distributing the movement on a larger area than the electrode and/or restricting epidermis deformation shows good results. However, more study is required to better understand the entire picture that includes skin, underlying tissue, and mechanical and electrical behavior.

To conclude, our general recommendation is that for dry electrodes intended for wearable systems, it is better to use soft padding behind the electrode for buffering vertical and lateral motion and to reduce pressure changes under the electrode due to body movement and muscular contraction as we also observed in our previous paper [8]. Moreover, an electrode support structure design that distributes motion over an area reaching beyond the electrode itself and stabilizes the epidermis around the electrode will further help to reduce the motion artifact.
